# Advancing Home Dialysis in Saudi Arabia: Challenges, Opportunities, and Future Directions

**DOI:** 10.7759/cureus.112477

**Published:** 2026-07-11

**Authors:** Hatem A Alnasser, Mohammad S Alsuhaibani, Yousef A Alsamil

**Affiliations:** 1 Department of Nephrology, King Saud University, Riyadh, SAU; 2 Department of Internal Medicine, Majmaah University, Riyadh, SAU

**Keywords:** end-stage renal disease, healthcare policy, home dialysis, peritoneal dialysis, saudi arabia

## Abstract

For individuals with end-stage renal disease, home dialysis offers a better clinical outcome, increased convenience, and an enhanced quality of life than in-center hemodialysis. It encompasses peritoneal dialysis and home hemodialysis, both of which allow patients to manage their treatment within their own homes, reducing dependency on hospital-based care. In Saudi Arabia, despite its recognized benefits, the adoption of home dialysis remains relatively low. Multiple factors, including infrastructure limitations, lack of awareness, and financial considerations, contribute to this challenge. This review explores the current landscape of home dialysis in Saudi Arabia, analyzing barriers to its widespread implementation, existing healthcare policies, technological advancements, and potential strategies to encourage broader adoption. By addressing key challenges such as patient education, provider training, and financial incentives, this review highlights actionable pathways for integrating home dialysis more effectively within the Saudi healthcare system. Enhancing home dialysis utilization could significantly improve healthcare outcomes and patient autonomy while reducing the burden on hospital resources. The review underscores the need for a collaborative approach among healthcare providers, policymakers, and stakeholders to ensure sustainable growth in home dialysis services in the Kingdom of Saudi Arabia.

## Introduction and background

Introduction

Chronic kidney disease (CKD) poses a significant healthcare burden in Saudi Arabia, with a rising prevalence of end-stage renal disease (ESRD) necessitating long-term dialysis or kidney transplantation [1]. While in-center hemodialysis (HD) is the dominant modality, home dialysis offers numerous advantages, including enhanced patient autonomy, fewer hospital visits, and improved clinical outcomes. However, its adoption remains suboptimal due to various logistical, cultural, and systemic barriers [2]. Home dialysis encompasses two principal modalities: peritoneal dialysis (PD) and home HD. Peritoneal dialysis, including continuous ambulatory peritoneal dialysis (CAPD), utilizes the patient’s peritoneal membrane for solute and fluid exchange and can be performed independently at home. Home HD involves the use of HD equipment in the home setting following appropriate patient and caregiver training. This review aims to assess the state of home dialysis in Saudi Arabia, identifying challenges and proposing strategies to enhance its utilization.

Literature search strategy

A narrative literature review was conducted to evaluate the current status, challenges, opportunities, and future directions of home dialysis in Saudi Arabia. Relevant literature was identified through electronic searches of PubMed and Google Scholar for articles published between January 2015 and December 2025. Search terms included “home dialysis,” “home hemodialysis,” “peritoneal dialysis,” “Saudi Arabia,” “chronic kidney disease,” “end-stage renal disease,” “renal replacement therapy,” “dialysis policy,” “caregiver support,” and “nephrology education.”

Additional publications were identified through manual review of reference lists from relevant articles. Studies addressing home dialysis utilization, barriers to adoption, implementation strategies, healthcare policy, patient outcomes, caregiver support, nephrology training, and healthcare delivery systems were prioritized. Preference was given to studies conducted in Saudi Arabia and comparable healthcare settings, while relevant international literature was included to provide additional context. The findings were synthesized narratively to provide an overview of the current evidence on home dialysis in Saudi Arabia.

Current status of home dialysis in Saudi Arabia

Overview of CKD and ESRD Prevalence in Saudi Arabia

CKD happens to be one of the global health states that currently has millions of patients worldwide suffering from it. The disease has many impacts, which include the quality of life, both physical and psychosocial well-being, costs or financial burden, mortality, and many more [3]. According to a study done in the year 2017, it was reported that almost 9.1% of the worldwide population is affected by CKD, which accounts for approximately 700 million cases [3]. Besides that, the study indicated that there had been a rise of 29.3% in the overall prevalence of CKD since 1990; however, the age-standardized prevalence did not change [3].

In Saudi Arabia, each area under the Ministry of Health manages its healthcare services and programs. Therefore, differences in prevalence and trends of CKD among these areas may be useful to health policymakers in specifically directing interventions and resource allocation. The population is predominantly young, with a significant proportion under 30 years of age. The Saudi Center for Organ Transplantation reported in 2019 that the total number of ESRD patients reported in Saudi Arabia was 28,256 (Table 1) [1].

**Table 1:** Global and Saudi Arabian prevalence of chronic kidney disease (CKD) and end-stage renal disease (ESRD) References: [1,3]

Statistic	Value	Source
Global CKD prevalence (2017)	9.1% (~700 million cases)	Study (2017) [3]
Increase in CKD prevalence since 1990	29.3%	Study (2017) [3]
Age-standardized CKD prevalence change	No significant change	Study (2017) [3]
Total ESRD cases in Saudi Arabia (2019)	28,256	Saudi Center for Organ Transplantation (2019) [1]

Causes of CKD That Lead to Dialysis

**Figure 1:**
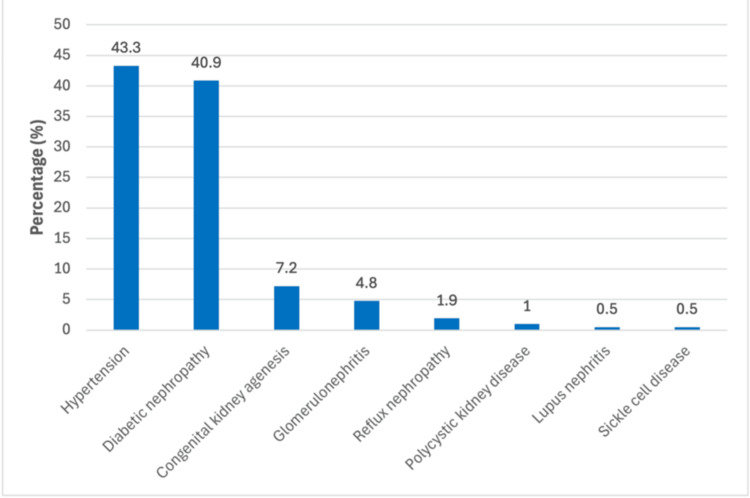
Causes of chronic kidney disease (CKD) leading to dialysis. Reproduced from reference [4] under a Creative Commons Attribution License CC-BY 4.0.

From July 6 to September 10, 2023, a cross-sectional descriptive study on CKD was conducted at the Al-Jaber Dialysis Center in the Al-Ahsa region of Saudi Arabia. High blood pressure (43.3%) and diabetic nephropathy (40.9%) were found to be the main causes of chronic kidney disease (CKD), which results in ESRD dialysis among patients [4,5]. Polycystic kidney disease (1%), unknown causes (4.8%), reflux nephropathy (1.9%), congenital kidney agenesis (7.2%), lupus nephropathy (0.5%), and sickle cell illness (0.5%) were less frequent causes (Table 2, Figure 1) [4].

**Table 2:** Causes of chronic kidney disease (CKD) leading to dialysis Reference: [4]

Cause	Percentage (%)
High blood pressure	43.3%
Diabetic nephropathy	40.9%
Polycystic kidney disease	1.0%
Unknown cause	4.8%
Reflux nephropathy	1.9%
Congenital kidney agenesis	7.2%
Lupus nephropathy	0.5%
Sickle cell illness	0.5%

## Review

Nephrologists′ services

A popular renal replacement technique, home HD, has been demonstrated to offer a number of advantages to both the patient and the healthcare system. In Saudi Arabia, however, home HD initiatives have not gained much traction. Through a survey-based cross-sectional study, we sought to investigate the opinions of Saudi Arabian adult nephrology consultants regarding the possible use of home HD [2]. The survey was sent via email to all adult nephrology consultants in Saudi Arabia registered with the Saudi Center of Nephrology and Organ Transplantation. Of the 236 consultants who were invited to participate in the research, 151 (64%) agreed to be a part of the study (Table 1). "Home HD is when a patient receives appropriate training and can perform their HD sessions by themselves in their home," stated half of the respondents on home HD. Eighty-one (54%) of the specialists admitted to never having managed a patient on HD during their nephrology training [2].

**Table 3:** Survey results on nephrologists' perspectives and barriers to home hemodialysis in Saudi Arabia HD: hemodialysis Reference: [2]

Parameter	Findings
Total invited nephrology consultants	236
Participants who agreed to the study	151 (64%)
Consultants defining home HD as patient-led dialysis at home	75 (49.6%)
Consultants who never managed a home HD patient during training	81 (54%)
Consultants acknowledging home HD advantages over in-center HD	108 (71.5%)
Consultants indicating no significant barriers to home HD in Saudi Arabia	120 (79.5%)
Consultants working in centers without an approved nephrology training program	60 (40%)
Perceived major barriers to home HD	Patient resistance, nephrologists' low motivation and experience, lack of managerial support, facilities, and nursing support

**Figure 2:**
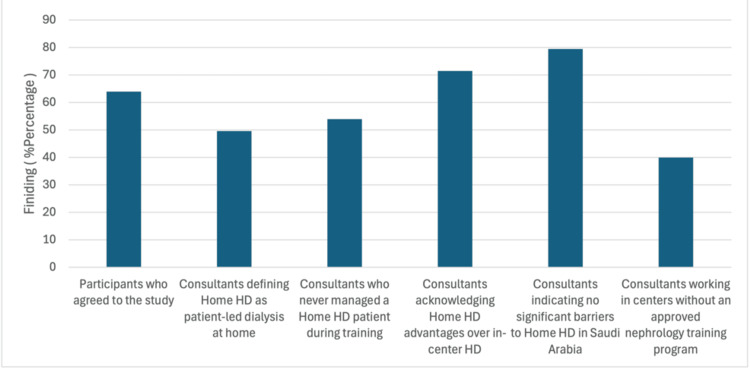
Nephrology consultants’ perspectives on home HD HD: hemodialysis Reference: [2]

More than 70% of participants confirmed that home HD has more advantages than in-center HD and poses no feasible barrier to Saudi Arabia (Figure 2) [2]. Notably, although 40% of the remaining participants worked in centers that did not have an approved nephrology training program, the vast majority felt that the local educational initiative did not impart sufficient knowledge to trainees about home HD [2]. By far, the major barriers that inhibit home dialysis use in Saudi Arabia include patient resistance, nephrologists’ low motivation and experience, and lack of managerial support, facilities, and nursing support [2].

Barriers to home dialysis adoption

**Figure 3:**
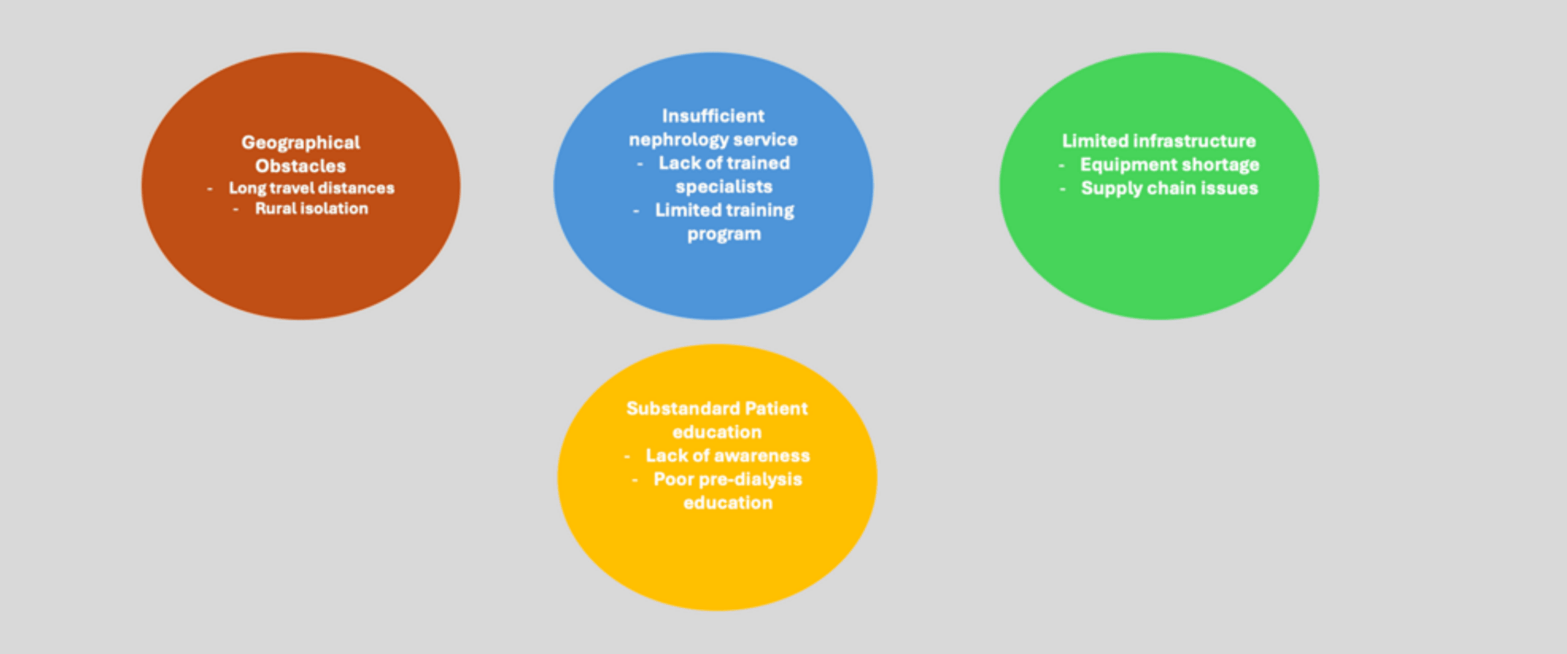
Major barriers to home dialysis adoption Reference: [2]

Chronic kidney disease and chronic renal failure, which require renal replacement therapy, are serious worldwide health concerns. One popular RRT technique is hemodialysis [4]. However, geographic obstacles and a lack of nephrology services make it difficult for patients in rural areas to receive hemodialysis (Figure 3).

Geographical Obstacles

Patients are allocated to a dialysis treatment facility near their home because there are typically enough dialysis centers in developed areas [4]. In impoverished areas, the opposite is true. To accommodate the rising demand, Saudi Arabia has 278 hemodialysis facilities with 8,165 dialysis machines [4].

However, the high cost of offloading and the challenge of traveling long distances for clinical appointments may prevent individuals from rural areas from accessing their preferred RRT option [4]. Additionally, residents of remote areas face additional system-level obstacles to the provision of healthcare, such as unequal physician supply, geographic isolation, and lower income, in addition to having to travel great distances to receive specialized medical care [4].

Along with such transportation issues, there are several systemic barriers that rural patients face in healthcare regarding renal replacement therapy (RRT). Among these barriers are unequal distribution of healthcare professionals, limited availability of nephrologists, and general shortages in medical resources [4]. Income levels are lower among rural populations, which also contributes to the difficulty of affording out-of-pocket healthcare costs and subsequently aggravates the disparities in treatment access [6].

Delay in referrals for kidney disease management, including both dialysis and transplantation, is another vital issue [7]. Patients in remote areas may have to wait a long time before a treatment option is evaluated just because of the inefficiency of coordination in healthcare services. Delays not only mean that such patients will not have access to dialysis but also narrow down their chances of being considered for life-saving kidney transplants. These geographical barriers result in increased morbidity and mortality among end-stage renal disease patients, and targeted strategies need to be put in place to improve accessibility to dialysis in rural and underserved areas.

Insufficient Nephrology Services** **

Lack of nephrology training and expertise hampers patient education and support for home dialysis, especially when very few nephrologists are trained in home dialysis programs [2,6]. Some centers lack accredited nephrology training programs, and many nephrologists learn little about home dialysis patients in their training [8]. Some nephrologists may be unwilling to recommend or undertake home dialysis programs on account of their inexperience.

Scarcity of nephrologists and specialized dialysis staff: Saudi Arabia is facing a growing burden due to CKD and ESRD, which is increasing the demand for nephrology services. Despite spending on healthcare infrastructure expansion, the number of nephrologists remains low and is unable to meet the ever-escalating patient population [8]. Shortcomings of this kind are very pronounced in rural and underprivileged areas in the much-needed access to nephrology specialists. In most cases, in the absence of enough nephrology knowledge, patients would not receive pre-dialysis comprehensive counseling, nor would they be conversant about home dialysis options.

Besides nephrologists, there are also very few dialysis-trained nurses and auxiliary healthcare personnel. There is a serious extra burden on the provision of home dialysis since lots of training is required for both patients and caregivers to deliver treatment safely and effectively [7]. Home-based dialectics are not optimized in many health care establishments, even where there are in-center hemodialysis. This is a sort of demotivational setup for patients and healthcare practitioners, and it aims to punish those seeking home-based modalities.

Limited Infrastructure and Resources

The lack of specialized facilities and equipment for home dialysis is a deterrent to operational adoption [2]; there will be no one who would allow for adequate training and support staff. Most hospitals do not have CKD clinics and multidisciplinary activities necessary for comprehensive health care for patients with kidney disease, as well as peritoneal dialysis units.

Inadequate availability of home dialysis equipment and supplies: The unavailability of home dialysis machinery, the availability, and distribution of the necessary equipment and consumables for dialysis, is one of the major barriers to home dialysis acceptance in Saudi Arabia [2]. Whereas in-center hemodialysis has machines, dialysates, and other supplies available in good time, home dialysis patients rely on an uninterrupted supply chain to provide these materials at home. Higher importation costs of dialysis machines, water purification systems, and dialysis fluids are another challenge leading to limited access to home dialysis.

Transport delays further disrupt treatment schedules in some outlying and underserved regions, putting the patients at high health risk [8]. Also, the maintenance cost of home dialysis systems, such as water treatment systems for home hemodialysis, often becomes a financial burden that many patients and families cannot support.

Insufficient home dialysis training centers: For effective administration of home dialysis, training of patients and caregivers, both in theory and practice, is important [9]. Very few centers exist in Saudi Arabia to train patients for home dialysis, denying them the opportunity for adequate education and clinical practice. Most training provided in health institutions is for inpatients on hemodialysis, leaving almost no time for structured educational programs for home therapies.

Substandard Patient Education and Support

The absence of pre-dialysis education and psychosocial support in patients suffering from CKD results in reluctance on the part of patients to consider home dialysis or to effectively manage it [9]. Patients are lacking in knowledge and skills concerning self-managing their dialysis at home, which ultimately causes complications and poor outcomes.

Lack of awareness about home dialysis options: The lack of awareness about home dialysis among patients and caregivers ranks as one of the main reasons for low adoption rates in treating CKD patients in Saudi Arabia [10]. Many patients suffering from CKD and ESRD are not adequately educated about home dialysis as a significant treatment option. Instead, in-center hemodialysis is typically portrayed as the standard of care or the option of choice.

The provision of education to patients about dialysis modalities lies primarily in the hands of the healthcare providers and nephrologists. However, due to the patient loads within Saudi dialysis centers, nephrologists do not have enough time for comprehensive counseling on home dialysis. Hence, patients miss their opportunities to make informed choices about treatment.

Pre-dialysis education programs in place have been inadequate: Pre-dialysis education helps CKD patients to understand their treatment options pre-ESRD better. In many developed systems, structured pre-dialysis programs educate patients about lifestyle changes, dialysis options, and transplantation through the application of theory and skill in practice. However, these programs are either lacking or implemented inconsistently in different hospitals and dialysis centers across Saudi Arabia [2,11].

In the absence of pre-dialysis education, a lot of patients are put on in-center hemodialysis without consideration of other options, where home dialysis may have been more suitable and convenient. Educating these patients at that early stage would enable them and their families to prepare well to manage home dialysis, hence improving adoption rates [12,13].

For example, assisted home hemodialysis gives people living with kidney failure the chance to receive their treatment in the comfort of their own home, with the support of a trained nurse or caregiver. It can make a big difference in their daily lives-offering more freedom, fewer trips to the hospital, and a greater sense of control. To help patients feel confident and supported, education should be personal and accessible. This can include simple, easy-to-understand materials, friendly one-to-one sessions with nurses, and community talks that answer questions and ease worries. The goal is to make patients feel safe, informed, and empowered in their care.

Lack of adequate training for patients and caregivers: Home dialysis depends on providing patients and caregivers with adequate hands-on training related to performing the various dialysis procedures, dealing with complications, and preventing, controlling, and managing infection. However, unfortunately, Saudi Arabia still has a shortage of dedicated training centers for home dialysis [1,2].

Many hospitals focus their energies on in-center hemodialysis and devote little to almost no time to home dialysis training [7]. When home dialysis training is conducted, it tends to provide too little, too fast, or offer hardly any follow-up support. Such loopholes in training lead to increased numbers of patients opting for in-center treatment in Saudi Arabia since they do not have the confidence to manage home dialysis on their own. Caregivers are equally important in home dialysis, especially when elderly or disabled patients are involved. They need training, as well as emotional support, in order for them not to feel overwhelmed and turn down the responsibility of aiding in home dialysis.

Administrative and Organizational Barriers

This creates more logistical challenges for home dialysis programs because of the absence of administrative support and the interrelationship between various healthcare providers [14]. Furthermore, the absence of specific guidelines and protocols for home dialysis hinders such practices. These impediments consist of inefficient bureaucratic procedures, insufficient policy support, and fragmented coordination among relevant participants in healthcare, all resulting in the underutilization of home dialysis as a viable renal replacement therapy (RRT) option.

Inefficient healthcare policies and regulations: Confusion reigns at the administrative level, which is one of the main barriers to home dialysis adoption in Saudi Arabia, where policies are either undeveloped or unclear and offer no support for home dialysis as a treatment option worthy of consideration [15]. In support of in-center hemodialysis, the systems are very much supportive, while home dialysis policies are far behind. As a whole, such policies do not equip hospitals or medical practitioners with sufficient incentives to recommend such home therapies.

Further, heavy-handed regulatory requirements for home setup, equipment approval, and training might dissuade the provider from working with home dialysis programs. The lack of standardized national guidelines for patient selection, training, and follow-up care creates additional confusion in the situation and leads to variations in service delivery across different regions.

Bureaucratic bottlenecks in equipment procurement and distribution: Home dialysis equipment-for example, dialysis machines, consumables, and water treatment systems-is prone to lengthy delays in availability due to bureaucratic barriers in procurement and distribution. Lengthy approval processes, import restrictions, and supply chain-related inefficiencies lead to irregular access to essential supplies for dialysis, especially in rural and remote areas. Budgetary constraints could also place additional obstacles in allocating resources to home dialysis programs [12]. In many situations, in-center hemodialysis is prioritized over home-based dialysis by governmental or private hospitals, thus reducing access to home-based treatment options even more.

Fragmented coordination between healthcare providers: The effective functioning of home dialysis programs requires continuous coordination among nephrologists, dialysis nurses, home healthcare providers, and supply chain managers. Fragmentations within the healthcare system in Saudi Arabia usually lead to miscommunication, delays in treatment initiation, and poor patient follow-ups [16]. For instance, there may be delays in referrals for home dialysis for some patients due to the lack of communication between primary care physicians and nephrologists. In addition, the absence of linkage between hospitals, dialysis centers, and home care providers through integrated electronic medical records (EMRs) makes it hard to keep track of patient progression and maintain continuity in care [17].

Opportunities and future directions

**Figure 4:**
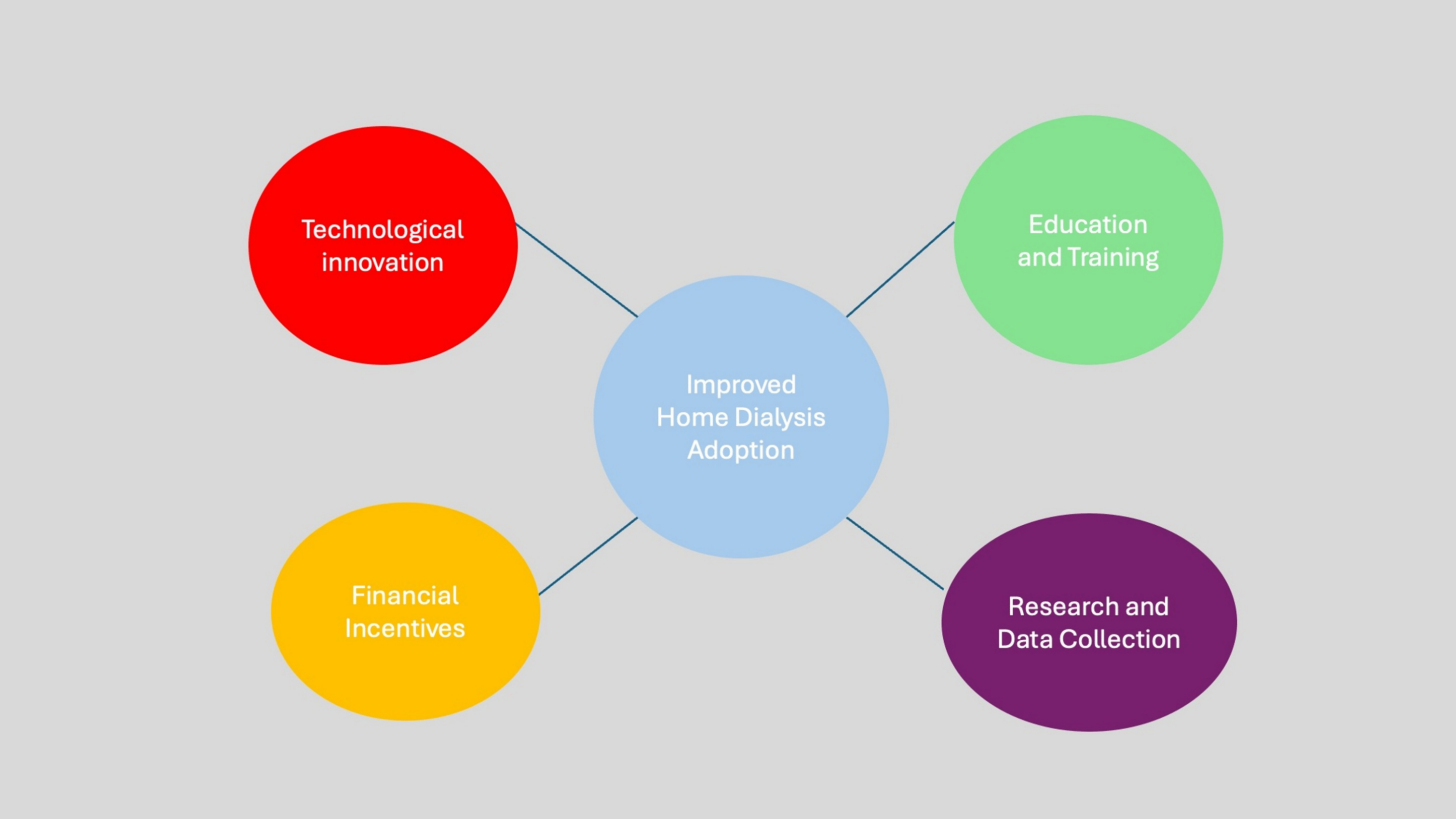
Key opportunities and future directions for improving home dialysis adoption Reference: [11]

Home hemodialysis provides patients and their caregivers with the opportunity to perform regular hemodialysis treatments in the comfort of their own homes. This modality offers numerous advantages, including greater convenience, improved quality of life compared with in-center hemodialysis, and reduced dependence on hospital-based care. Several opportunities exist to facilitate the establishment and expansion of home hemodialysis programs in the future. These include enhancing education and training for healthcare providers, patients, and caregivers, ensuring adequate financial support and resource allocation, and promoting the adoption of technological innovations in home hemodialysis (Figure 4).

Technological Innovations

The last decade has seen little innovativeness in nephrology, particularly when compared to major fields of medicine such as cardiovascular medicine or oncology (Technology Roadmap in Hemodialysis Therapy to Address the Unmet Needs) [13]. Transplantation is limited in itself. Despite promising distant applications for portable machines, wearable artificial kidneys and bio-artificial kidneys remain far-fetched and slow in their implementation for hemodialysis therapy.

The Hemodialysis Center of Al Salam Hospital is among the largest in the Eastern Province of the Kingdom of Saudi Arabia, providing advanced dialysis services at par with the highest potentials and standards set for health quality internationally and locally. These advanced facilities in medical hemodialysis devices cater to a wide spectrum of cases of kidney diseases and comprise a large team of multidisciplinary health professionals such as nephrologists, dialysis nurses, renal dietitians, renal pharmacists, and biomedical engineers, working in collaboration with urologists, endocrinologists, cardiologists, surgeons, and other specialists to provide effective care to patients suffering from kidney diseases.

Projects to Educate and Train

Programs targeted at education can increase awareness among patients and caregivers about the advantages and practicalities of home dialysis [16]. Training programs can build the confidence and competence of healthcare providers in managing home dialysis patients. Thus, home dialysis education should be embedded within medical and nursing curricula as a way of equipping future healthcare professionals to support this treatment modality.

Financial Incentives

Expanding government subsidies and insurance coverage for home dialysis can leverage even more patients to adopt home therapies [13]. Better public-private partnerships will lead to an increase in access to home dialysis infrastructure, equipment, and support services [18]. Create financial incentives for hospitals and clinics concerning the promotion of home dialysis, and probably turn attention to patient-centered care and sustainability in health delivery.

Research and Data Collection

By implementing research studies with enormous sample sizes on the cost-effectiveness, benefit analysis, and patient outcomes of home dialysis in Saudi Arabia, we are rendering timely evidence to policymakers [19]. Setting up a national registry for home dialysis patients could identify barriers and trends to evaluate impacts [20]. Promoting collaborative research initiatives between local institutions and international entities would enhance the flow of knowledge and best practices.

## Conclusions

Home hemodialysis in Saudi Arabia could be programmed for a multi-pronged approach. Improved education, supportive policy, and new technology developments would all be part of this last approach. There is an evident gap between a high incidence of in-center hemodialysis and a very low uptake of home dialysis. This demonstrates the burning need for interventions to be implemented strategically. Sizeable potential for improvement can be unlocked through addressing barriers and capitalizing upon available opportunities in enhancing the dialysis care framework within Saudi Arabia so that patients suffer less in terms of quality of life and health care outcomes. Creating awareness among patients and health care practitioners, in close partnership with research and investment by the governments in technology, would be a significant leap towards home dialysis therapy adoption.
